# Evaluation of WGS-subtyping methods for epidemiological surveillance of foodborne salmonellosis

**DOI:** 10.1186/s42522-020-00016-5

**Published:** 2020-07-06

**Authors:** Manal Mohammed, Salina Thapa

**Affiliations:** School of Life Sciences, College of Liberal Arts and Sciences, University of Westminster, London, UK

**Keywords:** *Salmonella*, WGS, Subtyping, SNP-typing, Prophage profile, CRISPR typing, MLST, rMLST, wgMLST, cgMLST

## Abstract

**Background:**

Salmonellosis is one of the most common foodborne diseases worldwide. Although human infection by non-typhoidal *Salmonella* (NTS) *enterica subspecies enterica* is associated primarily with a self-limiting diarrhoeal illness, invasive bacterial infections (such as septicaemia, bacteraemia and meningitis) were also reported. Human outbreaks of NTS were reported in several countries all over the world including developing as well as high-income countries. Conventional laboratory methods such as pulsed field gel electrophoresis (PFGE) do not display adequate discrimination and have their limitations in epidemiological surveillance. It is therefore very crucial to use accurate, reliable and highly discriminative subtyping methods for epidemiological characterisation and outbreak investigation.

**Methods:**

Here, we used different whole genome sequence (WGS)-based subtyping methods for retrospective investigation of two different outbreaks of *Salmonella* Typhimurium and *Salmonella* Dublin that occurred in 2013 in UK and Ireland respectively.

**Results:**

Single nucleotide polymorphism (SNP)-based cluster analysis of *Salmonella* Typhimurium genomes revealed well supported clades, that were concordant with epidemiologically defined outbreak and confirmed the source of outbreak is due to consumption of contaminated mayonnaise. SNP-analyses of *Salmonella* Dublin genomes confirmed the outbreak however the source of infection could not be determined. The core genome multilocus sequence typing (cgMLST) was discriminatory and separated the outbreak strains of *Salmonella* Dublin from the non-outbreak strains that were concordant with the epidemiological data however cgMLST could neither discriminate between the outbreak and non-outbreak strains of *Salmonella* Typhimurium nor confirm that contaminated mayonnaise is the source of infection, On the other hand, other WGS-based subtyping methods including multilocus sequence typing (MLST), ribosomal MLST (rMLST), whole genome MLST (wgMLST), clustered regularly interspaced short palindromic repeats (CRISPRs), prophage sequence profiling, antibiotic resistance profile and plasmid typing methods were less discriminatory and could not confirm the source of the outbreak.

**Conclusions:**

Foodborne salmonellosis is an important concern for public health therefore, it is crucial to use accurate, reliable and highly discriminative subtyping methods for epidemiological surveillance and outbreak investigation. In this study, we showed that SNP-based analyses do not only have the ability to confirm the occurrence of the outbreak but also to provide definitive evidence of the source of the outbreak in real-time.

## Introduction

Foodborne salmonellosis is an important concern for public health. It is caused by the enteric pathogen *Salmonella enterica*, which includes more than 2600 serovars [1]. Human *Salmonella* infections are classically divided into diseases caused by typhoidal or non-typhoidal salmonella (NTS). Typhoid fever is caused by the human restricted *Salmonella enterica* serovars Typhi and Paratyphi [2]. Although non-typhoidal *Salmonella* (NTS) serovars, predominantly cause a self-limiting diarrhoeal illness they have adapted to cause invasive extra-intestinal disease known as invasive NTS (iNTS) which can result in bacteraemia and focal systemic infections [3, 4] . There are two licenced vaccines for prevention of typhoid fever however, they are not effective against NTS [5] moreover, management of iNTS illness is complicated by the emergence of multidrug resistant (MDR) strains [6]. *Salmonella* serovars responsible for typhoid fever kill over 250,000 humans per year [7] while non-typhoidal *Salmonella* (NTS) serovars responsible for diarrhoeal illness cause over 155,000 deaths annually [8]. Interestingly, NTS have adapted to cause febrile bacteraemia and serious systemic infections; it has been estimated that over 680,000 people die every year as a result of infection by invasive NTS (iNTS) [3]. *Salmonella* Typhimurium and *Salmonella* Dublin have been associated with systemic illness [4, 5]. Human outbreaks of *Salmonella* Typhimurium and *Salmonella* Dublin were reported in developed countries [9–11].

Conventional laboratory methods such as pulsed field gel electrophoresis (PFGE) do not usually provide adequate discrimination among outbreak and non-outbreak strains of *Salmonella enterica* and have their limitations in epidemiological surveillance, it is therefore crucial to use accurate, reliable and highly discriminative subtyping methods for epidemiological characterisation and outbreak investigation.

Here, we evaluate different whole genome sequence (WGS)-based subtyping methods (including single nucleotide polymorphism (SNP)-based cluster analysis, multilocus sequence typing (MLST), ribosomal MLST (rMLST), whole genome MLST (wgMLST), core genome MLST (cgMLST) as well as clustered regularly interspaced short palindromic repeats (CRISPRs), prophage sequence profiling, antibiotic resistance profile and plasmid typing) for retrospective investigation of two outbreaks of *Salmonella* Typhimurium and *Salmonella* Dublin that occurred in 2013 in UK and Ireland respectively [9, 12].

## Methods

### Retrospective analyses of the two outbreaks of *Salmonella* Typhimurium and *Salmonella* Dublin

We carried out retrospective investigation of a human outbreak of *Salmonella* Dublin that occurred in 2013 in Ireland [9] and another human outbreak of *Salmonella* Typhimurium occurred in 2013 in UK [12]. We included suspected food strains isolated from mayonnaise and raw-milk cheeses that can be linked to the outbreaks of *Salmonella* Typhimurium and *Salmonella* Dublin respectively. Non-outbreak strains were also included for comparison. Details of all *Salmonella* Dublin and *Salmonella* Typhimurium isolates analysed in this study are provided in supplementary Tables 1 and 2 respectively.

PFGE was of a limited value for the investigation of the outbreak of *Salmonella* Dublin [9] since all outbreak and non-outbreak isolates of *Salmonella* Dublin were indistinguishable by PFGE. Although multiple loci VNTR analysis (MLVA) was of value in discriminating the outbreak strains from an epidemiologically unrelated isolate in 2013 it was not able to provide a conclusive link between the outbreak strain and a historical isolate from 2011 (11F310) since all outbreak strains had the same MLVA pattern (3-6-1-10-2-3-12) and the historical isolate had similar MLVA pattern (3–6–1-10-2-3-11/12).

Despite the technical limitation of phage typing, it was of value for investigating the outbreak of *Salmonella* Typhimurium [12] and confirming that mayonnaise is the source of infection.

### *Denovo* assembly of WGS data of *Salmonella* Dublin and *Salmonella* Typhimurium strains

We carried out *denovo* assembly for the raw Fastq paired end (PE) reads for all *Salmonella* Dublin and *Salmonella* Typhimurium strains using two different assemblers including Velvet available at Centre for genomic epidemiology (CGE) (http://www.genomicepidemiology.org/) and SPAdes available at Enterobase (http://enterobase.warwick.ac.uk/). We then assessed the quality of the assembly for each strain was assessed using Quast assessment tool (http://quast.bioinf.spbau.ru/).

### SNP typing analyses of *Salmonella* Dublin and *Salmonella* Typhimurium outbreaks

SNP analysis was carried out using CSIPhylogeny (https://cge.cbs.dtu.dk/services/CSIPhylogeny/) where raw reads were mapped to reference sequences (strain LT2 of *Salmonella* Typhimurium; accession number: AE006468 and strain CT_02021853 of *Salmonella* Dublin; accession number: CP001144) using BWA software (http://bio-bwa.sourceforge.net). The depth at each mapped position was calculated using genomeCoverageBed, which is part of BEDTools (https://bedtools.readthedocs.io/en/latest/). High quality SNPs were called using mpileup which is part of SAMTools (http://samtools.sourceforge.net). Genome mappings were then compared and an alignment of the SNPs are then created by concatenating the SNPs. A maximum likelihood (ML) phylogenetic tree was then created based on the concatenated alignment of the high quality SNPs.

### Determination of MLST, rMLST, cgMLST and wgMLST of *Salmonella* Dublin and *Salmonella* Typhimurium strains

The assembled sequences of each strain were analyzed to detect the MLST, rMLST, cgMLST and wgMLST available at Enetrobase (http://enterobase.warwick.ac.uk/) and CGE (http://www.genomicepidemiology.org/).

### Determination of prophage sequence profiles in *Salmonella* Dublin *and Salmonella* Typhimurium genomes

Prophages were determined with the draft genomes generated by Velevt and SPAdes for all *Salmonella* Dublin and *Salmonella* Typhimurium strains using PHASTER (http://phaster.ca/).

We then used CSI phylogeny available at CGE (http://www.genomicepidemiology.org/) to construct a phylogenetic tree based on the SNPs of detected prophages. Phylogenetic trees were constructed using assembled genomes generated by Velvet and SPAdes assemblers to check if the assembly could affect the tree.

### Determination of CRISPRs within *Salmonella* Dublin and *Salmonella* Typhimurium strains

Spacers sequence within the draft genomes of all *Salmonella* Dublin and *Salmonella* Typhimurium strains were characterized using CRISPRFinder (http://crispr.i2bc.paris-saclay.fr/Server/).

### Determination of plasmids within *Salmonella* Dublin and *Salmonella* Typhimurium strains

We determined the plasmids within the draft genomes of all *Salmonella* Dublin and *Salmonella* Typhimurium strains using the plasmid database; PLSDB (https://ccb-microbe.cs.uni-saarland.de/plsdb/).

### In silico analyses of antibiotic resistance within *Salmonella* Dublin and *Salmonella* Typhimurium strains

We determined acquired antibiotic resistance genes and mutations within the draft genomes of all *Salmonella* Dublin and *Salmonella* Typhimurium strains using ResFinder (https://cge.cbs.dtu.dk/services/ResFinder/).

## Results

### WGS-based subtyping

#### SNP based cluster analyses

**Fig. 1:**
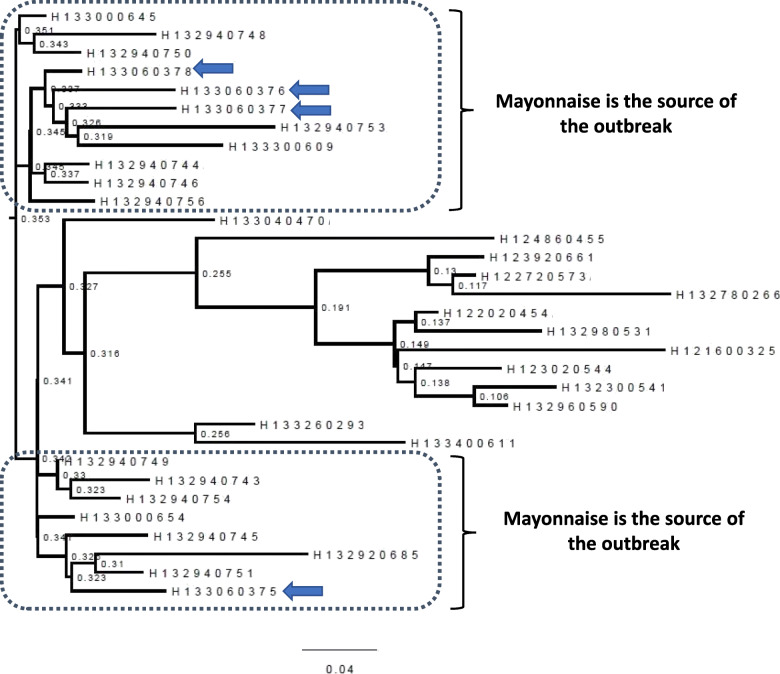
Maximum likelihood phylogenetic tree of *Salmonella* Typhiurium strains based on single nucleotide polymorphisms determined from whole genome sequences. The scale represents the number of nucleotide substitutions per site. Bootstrap support values, given as a percentage of 1000 replicates, are shown on the branches. The tree shows conclusively that myonaise (marked with arrows) is the source of outbreak

SNP based tree showed conclusively that the outbreak strains of *Salmonella* Typhimurium were grouped together in two clades and they are very closely related to strains isolated from mayonnaise (Fig. 1) confirming the source of outbreak is due to consumption of contaminated mayonnaise.

**Fig. 2:**
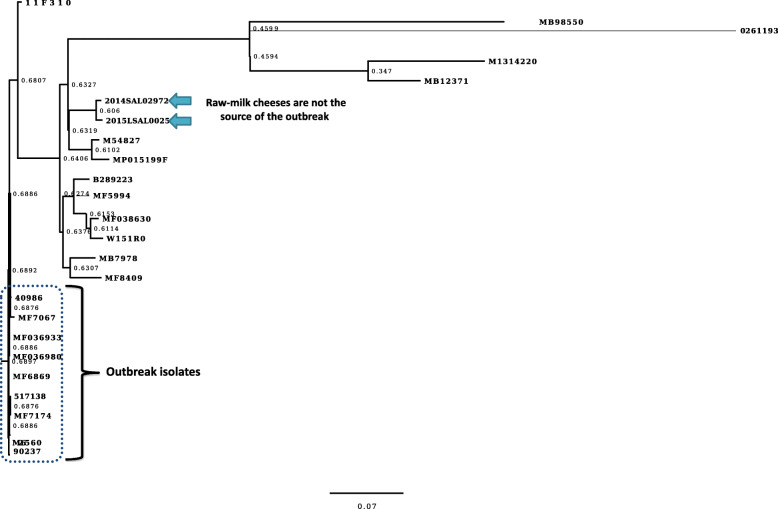
Maximum likelihood phylogenetic tree of *Salmonella* Dublin strains based on single nucleotide polymorphisms determined from whole genome sequences. The scale represents the number of nucleotide substitutions per site. Bootstrap support values, given as a percentage of 1000 replicates, are shown on the branches. All *Salmonella* Dublin isolates had indistinguishable pulsed-field gel electrophoresis profiles. Confirmed outbreak cases (*n* = 9) in October–November 2013 are grouped together in one cluster. However, the source of the outbreak could not be determined as outbreak isolates showed high genetic divergence to bacterial strains isolated from the raw-milk cheeses (marked with arrows) including isolate 2014SAL02972 from Morbier cheese (accession number; ERS2767809) and isolate 2015LSAL00258 from St. Nectaire cheese (accession number: ERS2767808)

The outbreak isolates of *Salmonella* Dublin were closely related to each other (Fig. 2) and distinct from the non-outbreak isolates that were not readily distinguishable by PFGE. However, the source of *Salmonella* Dublin outbreak could not be determined and outbreak isolates showed high genetic divergence from the raw-milk cheese isolates related to other outbreaks occurred in France [10].

#### MLST, rMLST, cgMLST and wgMLST

As illustrated in Table 1, all *Salmonella* Dublin strains including the outbreak and non-outbreak strains showed identical MLST (type 10). Interestingly, outbreak isolates of *Salmonella* Dublin displayed identical rMLST (type 1429) however, some of the non-outbreak strains showed the same rMLST. Moreover, the wgMLST was different among the outbreak strains however, the cgMLST was unique among outbreak strains and can easily separate the outbreak strain from the non-outbreak strains including the 2011 historical isolate (11F310).

**Table 1:** MLST, rMLST, cgMLST and wgMLST results of *Salmonella* Dublin outbreak and non-outbreak strains

Strain ID:	MLST:	rMLST:	cgMLST:	wgMLST:
**Outbreak strains:**				
902,637	10	1429	38,665	259,116
MF036933	10	1429	38,665	259,117
MF036980	10	1429	38,665	259,118
517,138	10	1429	38,665	259,121
MF6869	10	1429	38,665	259,127
M26560	10	1429	38,665	259,123
MF7067	10	1429	38,665	259,122
MF7174	10	1429	38,665	259,128
40,986	10	1429	38,665	259,126
**Non-outbreak strains:**				
MF038630	10	1429	38,666	259,131
M1314220	10	26,829	38,664	259,120
M54827	10	1429	38,667	259,129
MB12371	10	26,829	38,668	259,130
MF5994	10	92,451	38,669	259,145
MB7978	10	1429	38,670	259,133
B289223	10	1429	38,671	259,134
11F310	10	1429	38,655	259,135
MB98550	10	3696	38,657	259,142
MF8409	10	1429	38,658	259,139
W151R0	10	1429	38,659	259,140
B261193	10	92,450	38,660	259,141
MP015199F	10	1429	38,661	259,148
**Food isolates:**				
^a^2014LSAL02972	10	1429	230,922	283,421
^a^2015LSAL00258	10	96,856	146,469	283,422

Same results for MLST, rMLST, cgMLST and wgMLST were obtained from CGE and Enterobase using Velvet and SPAdes assemblers respectively.

^a^*Salmonella* Dublin strains isolated from raw milk cheeses related to other outbreaks occurred in France [10]

On the other hand, MLST, rMLST, cgMLST and wgMLST could not discriminate between the outbreak and non-outbreak strains of *Salmonella* Typhimurium as illustrated in Table 2.

**Table 2:** MLST, rMLST, cgMLST and wgMLST results of *Salmonella* Typhimurium outbreak and non-outbreak strains

Strain ID	MLST:	rMLST:	cgMLST:	wgMLST:
**Food strains:**				
^a^H133060375	19	1392	60,658	70,401
^a^H133060376	19	1392	60,660	70,402
^a^H133060377	19	1392	36,749	70,514
^a^H133060378	19	1392	60,661	70,403
**Outbreak strains:**				
H133000654	19	1392	36,749	70,398
H132940743	19	1392	36,749	70,404
H132940744	19	1392	60,662	70,405
H132940745	19	1392	60,663	70,406
H132940746	19	1392	36,749	70,431
H132940748	19	1392	60,683	70,432
H132940749	19	1392	36,749	70,433
H132940750	19	1392	60,684	70,439
H132940751	19	1392	60,685	70,440
H132940753	19	1392	61,002	70,834
H132940754	19	1392	36,754	70,835
H132940756	2392	1392	61,001	70,833
H133000645	19	1392	36,749	
H133300609	19	1392	36,749	70,944
H132300541	19	1391	36,751	70,951
**Non-outbreak strains:**				
H133260293	19	1392	71,438	84,026
H132780266	19	1391	71,450	84,040
H132960590	19	1391	36,751	84,041
H132920685	19	1392	36,763	84,076
H132980531	19	1391	36,774	87,971
H121600325	19	1391	20,224	87,972
H122720573	19	1391	20,848	87,973
H12320661	19	1391	20,882	87,974
H123020544	19	1391	20,711	87,975
H122020454	19	1391	21,310	88,017
H124860455	19	26,127	20,800	88,018
H133040470	19	1392	71,422	84,006
H1330400611	19	1392	71,438	84,025

^a^Strains of *Salmonella* Typhimurium isolated from mayonnaise

Same results for MLST, rMLST, cgMLST and wgMLST were obtained from CGE and Enterobase using Velvet and SPAdes assemblers respectively.

#### CRISPR typing

All *Salmonella* Dublin isolates including outbreak and non-outbreak strains harbour one CRISPR locus and we observed 3 to 5 unique spacers for CRISPR1 locus. Identical spacers were detected among the outbreak and non-outbreak strains as shown in Table 3.

**Table 3:** Number of spacers within CRISPR1 locus in all *Salmonella* Dublin strains analysed in this study

Strain ID:	Spacers No. (Velvet)	Spacers No. (SPAdes)
**Outbreak strains:**		
902,637	**5**	**5**
MF036933	**5**	**5**
MF036980	**5**	**5**
517,138	**4**	**5**
MF6869	**5**	**5**
M26560	**5**	**5**
MF7067	**4**	**5**
MF7174	**5**	**5**
40,986	**5**	**5**
**Non-outbreak strains:**		
MF038630	**5**	**5**
M1314220	**5**	**5**
M54827	**3**	**3**
MB12371	**5**	**5**
MF5994	**5**	**5**
MB7978	**5**	**5**
B289223	**5**	**5**
11F310	**5**	**5**
MB98550	**4**	**4**
MF8409	**5**	**5**
W151R0	**4**	**5**
B261193	**3**	**3**
MP015199F	**3**	**3**

Interestingly, the number of spacers in three isolates (517,138, MF7067 and W151R0) changed from (4 spacers) based on Velvet to (5 spacers) based on SPAdes.

All *Salmonella* Typhimurium isolates harbour 3 CRISPR loci. Identical spacers were detected among the outbreak and non-outbreak strains as shown in Table 4. There was no difference between the numbers of spacers using different assemblers.

**Table 4:** Number of spacers within CRISPRs loci in all *Salmonella* Typhimurium strains analysed in this study

Strain ID	Spacers No. (Velvet & SPAdes)		
**Food strains:**			
^a^**H133060375**	9	13	9
^a^**H133060376**	9	13	9
^a^**H133060377**	9	13	9
^a^**H133060378**	9	13	9
**Outbreak strains:**			
H133300609	9	13	9
H132940743	9	13	9
H132940744	9	13	9
H132940745	9	13	9
H132940746	9	13	9
H132940748	9	13	9
H132940749	9	13	9
H132940750	9	13	9
H132940751	9	13	9
H132940753	13	9	9
H132940754	9	13	9
H132940756	9	13	9
H133000645	9	13	9
H133000654	9	13	9
**Non-outbreak strains**			
H121600325	9	13	9
H122020454	9	13	9
H122720573	9	13	9
H123020544	9	13	9
H123920661	9	13	9
H124860455	9	13	9
H132780266	9	13	9
H132920685	9	13	9
H132960590	9	13	9
H132980531	9	13	9
H133040470	9	13	9
H133260293	9	13	9
H133400611	9	13	9

^a^Strains of *Salmonella* Typhimurium isolated from mayonnaise

#### Prophage sequence profiling

**Fig. 3:**
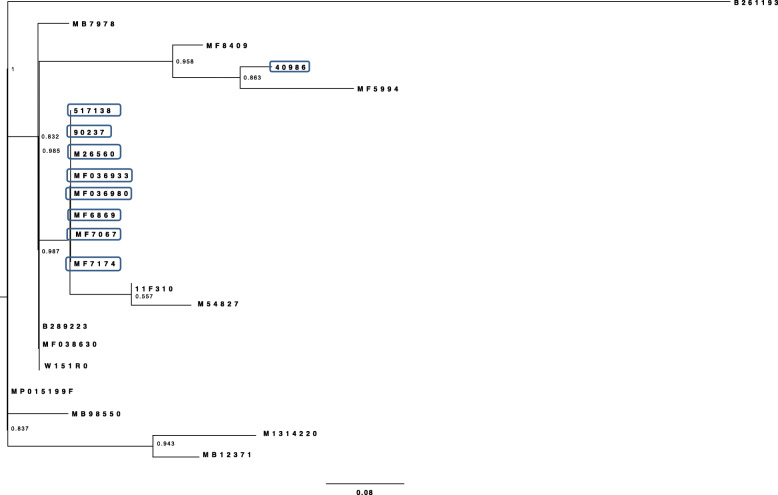
Maximum likelihood phylogenetic tree of *Salmonella* Dublin strains based on prophages SNPs using Velvet

**Fig. 4:**
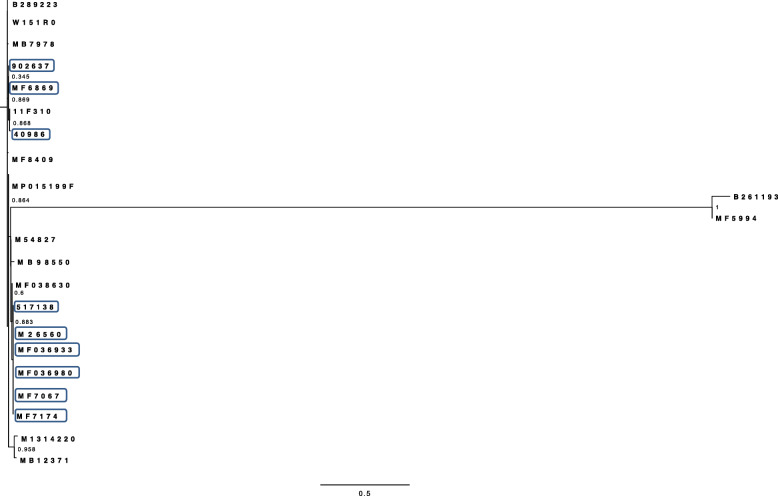
Maximum likelihood phylogenetic tree of *Salmonella* Dublin strains based on prophages SNPs using SPAdes

All *Salmonella* Dublin strains including the outbreak strains are lysogenic for three prophages (Gifsy_2, 118970_sal3 and RE_2010). However, phylogenetic analyses of *Salmonella* Dublin strains based on the SNPs of prophages showed that outbreak strains are intermixed with the non-outbreak strains based on velvet assembler (Fig. 3) and SPAdes assembler (Fig. 4).

All *Salmonella* Typhimurium genomes assembled by SPAdes revealed the presence of four prophages in all outbreak and non-outbreak strains including the three Salmonella prophages (Gifsy 2, RE-2010, and 118970_sal3) and the Edwardsiella specific phage (GF-2).

On the other hand, *Salmonella* Typhimurium genomes assembled by Velvet were lysogenic for two *Salmonella* specific prophages (Gifsy 2 and RE-2010). All strains except one outbreak isolate (H132940750) harbour *Salmonella* 118970_sal3 phage.

Interestingly, all strains harbour Edwardsiella GF-2 prophage except three outbreak isolates (H132940748, H133000645 and H133060376).

**Fig. 5:**
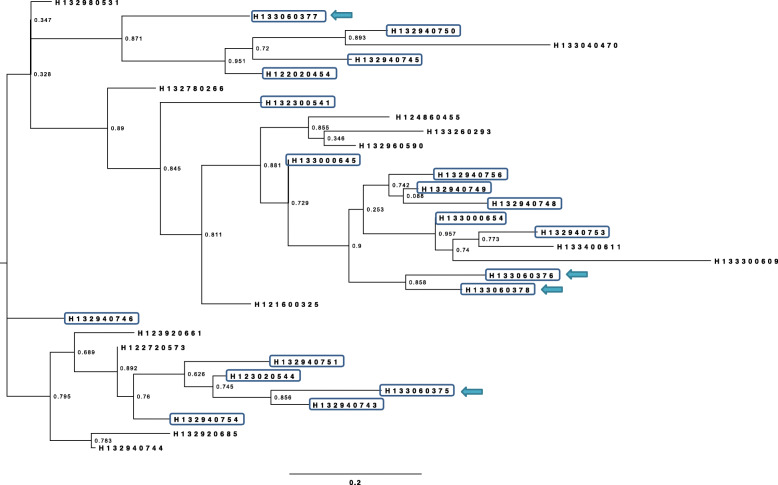
ML phylogenetic tree of *Salmonella* Typhimurium strains based on prophages SNPs using Velvet

**Fig. 6:**
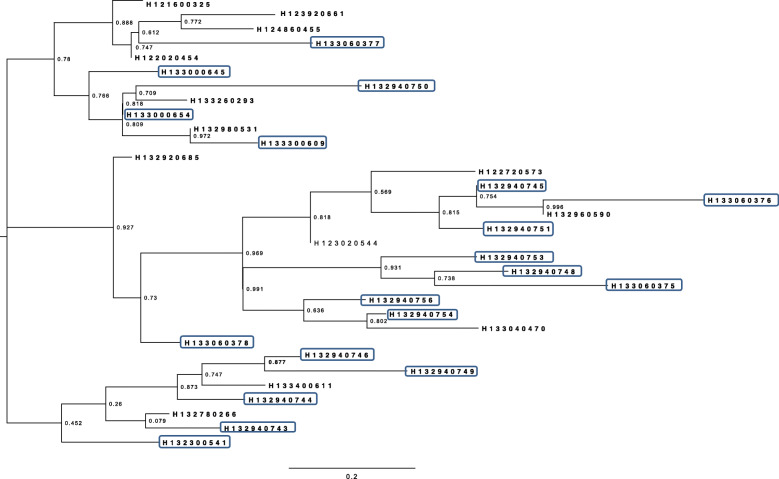
ML phylogenetic tree of *Salmonella* Typhimurium strains based on prophages SNPs using SPAdes

Phylogenetic analyses of *Salmonella* Typhimurium strains based on the SNPs of prophages showed that outbreak strains are intermixed with the non-outbreak strains using velvet assembler (Fig. 5) and using SPAdes assembler (Fig. 6).

#### Plasmid typing

All outbreak and non-outbreak strains of *Salmonella* Dublin harbour identical plasmid type (except three non-outbreak isolates; M1314220, MB12371 and B261193) as shown in Table 5.

**Table 5:** Distribution of plasmids among outbreak and non-outbreak strains of *Salmonella* Dublin

	pSA19992307 (NZ_CP030208)	pSE81–1705 (NZ_CP018654)	Plasmid: 4 (LN829404)	pATCC39184 (NZ_CP019180)	pSDU2-USMARC-69807 (NZ_CP032381)	Plasmid: 3 (NZ_LN868945)
**Outbreak strains:**						
**902,637**	Present	Present	Present	Absent	Absent	Absent
**MF036933**	Present	Present	Present	Absent	Absent	Absent
**MF036980**	Present	Present	Present	Absent	Absent	Absent
**517,138**	Present	Present	Present	Absent	Absent	Absent
**MF6869**	Present	Present	Present	Absent	Absent	Absent
**M26560**	Present	Present	Present	Absent	Absent	Absent
**MF7067**	Present	Present	Present	Absent	Absent	Absent
**MF7174**	Present	Present	Present	Absent	Absent	Absent
**40,986**	Present	Present	Present	Absent	Absent	Absent
**Non-outbreak strains:**						
**MF038630**	Present	Present	Present	Absent	Absent	Absent
**M1314220**	**Absent**	Present	Present	**Present**	Absent	Absent
**M54827**	Present	Present	Present	Absent	Absent	Absent
**MB12371**	**Absent**	Present	Present	**Present**	Absent	Absent
**MF5994**	Present	Present	Present	Absent	Absent	Absent
**MB7978**	Present	Present	Present	Absent	Absent	Absent
**B289223**	Present	Present	Present	Absent	Absent	Absent
**11F310**	Present	Present	Present	Absent	Absent	Absent
**MB98550**	Present	Present	Present	Absent	Absent	Absent
**MF8409**	Present	Present	Present	Absent	Absent	Absent
**W151R0**	Present	Present	Present	Absent	Absent	Absent
**B261193**	**Absent**	Present	Present	Absent	**Present**	Absent
**MP015199F**	Present	Present	Present	Absent	Absent	Absent
**Food strains:**						Absent
^a^2014LSAL02972	Present	Present	Absent	Absent	Absent	Present
^a^2015LSAL00258	Present	Present	Absent	Absent	Absent	Present

^a^*Salmonella* Dublin strains isolated from raw milk cheeses related to other outbreaks occurred in France [10]

Same plasmids were determined using Velvet and SPAdes assemblers.

All outbreak and non-outbreak isolates of *Salmonell*a Typhimurium harbour 3 plasmids (pATCC14028, plasmid: 4 and pSE81–1705) except the outbreak strain H133300609 which did not carry plasmid pATCC14028 but it harbours a different plasmid (pSLT_VNP20009) instead (Table 6).

**Table 6:** Distribution of plasmids among outbreak and non-outbreak strains of *Salmonella* Typhimurium

Strain ID	pATCC14028 (NZ_CP034231)	Plasmid: 4 (LN829404)	pSE81–1705 (NZ_CP018654)	pSLT_VNP20009 (NZ_CP008745)
**Food strains:**				
^a^**H133060375**	Present	Present	Present	Absent
^a^**H133060376**	Present	Present	Present	Absent
^a^**H133060377**	Present	Present	Present	Absent
^a^**H133060378**	Present	Present	Present	Absent
**Outbreak strains:**				
**H132300541**	Present	Present	Present	Absent
**H132940743**	Present	Present	Present	Absent
**H132940744**	Present	Present	Present	Absent
**H132940745**	Present	Present	Present	Absent
**H132940746**	Present	Present	Present	Absent
**H132940748**	Present	Present	Present	Absent
**H132940749**	Present	Present	Present	Absent
**H132940750**	Present	Present	Present	Absent
**H132940751**	Present	Present	Present	Absent
**H132940753**	Present	Present	Present	Absent
**H132940754**	Present	Present	Present	Absent
**H132940756**	Present	Present	Present	Absent
**H133000645**	Present	Present	Present	Absent
**H133000654**	Present	Present	Present	Absent
**H133300609**	**Absent**	Present	Present	**Present**
**Non-outbreak strains:**				
**H121600325**	Present	Present	Present	Absent
**H122020454**	Present	Present	Present	Absent
**H122720573**	Present	Present	Present	Absent
**H123020544**	Present	Present	Present	Absent
**H123920661**	Present	Present	Present	Absent
**H124860455**	Present	Present	Present	Absent
**H132780266**	Present	Present	Present	Absent
**H132920685**	Present	Present	Present	Absent
**H132960590**	Present	Present	Present	Absent
**H132980531**	Present	Present	Present	Absent
**H133040470**	Present	Present	Present	Absent
**H133260293**	Present	Present	Present	Absent
**H133400611**	Present	Present	Present	Absent

^a^Strains of *Salmonella* Typhimurium isolated from mayonnaise

#### Antibiotic resistance profile

All *Salmonella* Dublin isolates including the outbreak and non-outbreak strains are resistant to aminoglycosides due to the acquisition of the *aac(6′)-Iaa* gene. No mutations were detected against *gyrA* and *parC* genes in all isolates except one isolate (MF038630) that carried a non-synonyms mutation within the gyrase protein and it is associated with bacterial resistance to nalidixic acid (Table 7).

**Table 7:** In silico analyses results of antimicrobial resistance genes and mutations within all *Salmonella* Dublin strains

Strain ID:	Acquired antibiotic resistance genes:	Mutations in ***gyrA gene:***	Mutations in ***parC gene:***
**Outbreak strains:**			
**902,637**	Aminoglycoside (*aac(6′)-Iaa*)	Absent	Absent
**MF036933**	Aminoglycoside (*aac(6′)-Iaa*)	Absent	Absent
**MF036980**	Aminoglycoside (*aac(6′)-Iaa*)	Absent	Absent
**517,138**	Aminoglycoside (*aac(6′)-Iaa*)	Absent	Absent
**MF6869**	Aminoglycoside (*aac(6′)-Iaa*)	Absent	Absent
**M26560**	Aminoglycoside (*aac(6′)-Iaa*)	Absent	Absent
**MF7067**	Aminoglycoside (*aac(6′)-Iaa*)	Absent	Absent
**MF7174**	Aminoglycoside (*aac(6′)-Iaa*)	Absent	Absent
**40,986**	Aminoglycoside (*aac(6′)-Iaa*)	Absent	Absent
**Non-outbreak strains:**			
**MF038630**	Aminoglycoside (*aac(6′)-Iaa*)	Absent	**Present**
**M1314220**	Aminoglycoside (*aac(6′)-Iaa*)	Absent	Absent
**M54827**	Aminoglycoside (*aac(6′)-Iaa*)	Absent	Absent
**MB12371**	Aminoglycoside (*aac(6′)-Iaa*)	Absent	Absent
**MF5994**	Aminoglycoside (*aac(6′)-Iaa*)	Absent	Absent
**MB7978**	Aminoglycoside (*aac(6′)-Iaa*)	Absent	Absent
**B289223**	Aminoglycoside (*aac(6′)-Iaa*)	Absent	Absent
**11F310**	Aminoglycoside (*aac(6′)-Iaa*)	Absent	Absent
**MB98550**	Aminoglycoside (*aac(6′)-Iaa*)	Absent	Absent
**MF8409**	Aminoglycoside (*aac(6′)-Iaa*)	Absent	Absent
**W151R0**	Aminoglycoside (*aac(6′)-Iaa*)	Absent	Absent
**B261193**	Aminoglycoside (*aac(6′)-Iaa*)	Absent	Absent
**MP015199F**	Aminoglycoside (*aac(6′)-Iaa*)	Absent	Absent
**Food strains:**			
^a^2014LSAL02972	Aminoglycoside (*aac(6′)-Iaa*)	Absent	Absent
^a^2015LSAL00258	Aminoglycoside (*aac(6′)-Iaa*)	Absent	Absent

^a^*Salmonella* Dublin strains isolated from raw milk cheeses related to other outbreaks occurred in France [10]

All the *Salmonella* Typhimurium isolates of both the outbreak and non-outbreak group are resistant to aminoglycosides due to the acquisition of the “*aac(6′)-Iaa* gene”. No known mutations were detected against *gyrA* and *parC* (Table 8).

**Table 8:** In silico analyses results of antimicrobial resistance genes and mutations within all *Salmonella* Typhimurium strains

Strain ID	Acquired antibiotic resistance genes:	Mutations in ***gyrA gene:***	Mutations in ***parC gene:***
**Food strains:**			
^a^**H133060375**	Aminoglycoside (aac(6′)-Iaa)	Absent	Absent
^a^**H133060376**	Aminoglycoside (aac(6′)-Iaa)	Absent	Absent
^a^**H133060377**	Aminoglycoside (aac(6′)-Iaa)	Absent	Absent
^a^**H133060378**	Aminoglycoside (aac(6′)-Iaa)	Absent	Absent
**Outbreak strains:**			
H132940743	Aminoglycoside (aac(6′)-Iaa)	Absent	Absent
H132940744	Aminoglycoside (aac(6′)-Iaa)	Absent	Absent
H132940745	Aminoglycoside (aac(6′)-Iaa)	Absent	Absent
H132940746	Aminoglycoside (aac(6′)-Iaa)	Absent	Absent
H132940748	Aminoglycoside (aac(6′)-Iaa)	Absent	Absent
H132940749	Aminoglycoside (aac(6′)-Iaa)	Absent	Absent
H132940750	Aminoglycoside (aac(6′)-Iaa)	Absent	Absent
H132940751	Aminoglycoside (aac(6′)-Iaa)	Absent	Absent
H132940753	Aminoglycoside (aac(6′)-Iaa)	Absent	Absent
H132940754	Aminoglycoside (aac(6′)-Iaa)	Absent	Absent
H132940756	Aminoglycoside (aac(6′)-Iaa)	Absent	Absent
H133000645	Aminoglycoside (aac(6′)-Iaa)	Absent	Absent
H133000654	Aminoglycoside (aac(6′)-Iaa)	Absent	Absent
H133300609	Aminoglycoside (aac(6′)-Iaa)	Absent	Absent
**Non-outbreak strains:**			
H121600325	Aminoglycoside (aac(6′)-Iaa)	Absent	Absent
H122020454	Aminoglycoside (aac(6′)-Iaa)	Absent	Absent
H122720573	Aminoglycoside (aac(6′)-Iaa)	Absent	Absent
H123020544	Aminoglycoside (aac(6′)-Iaa)	Absent	Absent
H123920661	Aminoglycoside (aac(6′)-Iaa)	Absent	Absent
H124860455	Aminoglycoside (aac(6′)-Iaa)	Absent	Absent
H132780266	Aminoglycoside (aac(6′)-Iaa)	Absent	Absent
H132920685	Aminoglycoside (aac(6′)-Iaa)	Absent	Absent
H132960590	Aminoglycoside (aac(6′)-Iaa)	Absent	Absent
H132980531	Aminoglycoside (aac(6′)-Iaa)	Absent	Absent
H133040470	Aminoglycoside (aac(6′)-Iaa)	Absent	Absent
H133260293	Aminoglycoside (aac(6′)-Iaa)	Absent	Absent
H133400611	Aminoglycoside (aac(6′)-Iaa)	Absent	Absent

^a^Strains of *Salmonella* Typhimurium isolated from mayonnaise

## Discussion

Salmonellosis is one of the most common foodborne diseases worldwide and has been associated with high morbidity and mortality rates. It is estimated that over 680,000 humans throughout the world are killed each year by iNTS. The most predominant iNTS serovars are Typhimurium, Enteritidis and Dublin [13, 14]. It is therefore very crucial to use accurate, reliable and highly discriminative subtyping methods for epidemiological surveillance and outbreak investigation.

Although PFGE is considered as current gold standard for all *Salmonella* serotypes, it has its limitations moreover, variation between laboratories has been reported when identifying the source of infection and discriminating between the outbreak and non-outbreak isolates [15].

Other phenotypic tools such as phage typing and antimicrobial resistance profiling have been crucial in the outbreak investigations [15, 16]. Furthermore, MLVA has been used to distinguish between genetically closely related strains and trace back the sources of disease outbreaks related to food [15, 17].

Genotypic approaches have ameliorated the methods for carrying out outbreak investigation and epidemiological surveillance [18]. The advent of whole genome sequencing (WGS) has opened the possibilities to enhance the typing approaches for outbreak investigation and epidemiological surveillance. In our study, WGS data have been analyzed to test the suitability of different approaches as subtyping tool for *Salmonella enterica* surveillance. We therefore carried out retrospective investigation of two different outbreaks of *Salmonella* Typhimurium and *Salmonella* Dublin that occurred in 2013 in UK and Ireland respectively [6, 19] using different WGS-subtyping methods.

In this study, single nucleotide polymorphism (SNP)-based cluster analysis of *Salmonella* Typhimurium genomes revealed well supported clades, that were concordant with epidemiologically defined outbreak and confirmed the source of outbreak is due to consumption of contaminated mayonnaise. Although SNP-analyses of *Salmonella* Dublin genomes confirmed the outbreak, however the source of infection could not be determined.

On the other the WGS-subtyping methods including MLST, rMLST, wgMLST, cgMLST showed limited discrimination for the outbreak and non-outbreak isolates of *Salmonella* Typhimurium strains. However, cgMLST defined the genetic relatedness among *Salmonella* Dublin isolates more precisely and confirmed there is no relation among the 2013 outbreak isolates and the 2011 historical isolate (11F310) of *Salmonella* Dublin.

It was reported that MLST might not be the most suitable epidemiological tool [20] but it is best for analyzing the genetic diversity of the strain and analyze the core and conserved genes of pathogens that are of public importance.

The cgMLST bridges the classic MLST with the novel WGS-based approach since it combines the discriminatory power of MLST with large-scale data obtained from WGS enabling to exploit a considerable number of gene targets throughout the bacterial genome which would maximize the quality and resolution for surveillance and research works.

A recent study showed that cgMLST has shown the robustness of cgMLST as a tool to investigate multi-country outbreak of *Salmonella* Enteritidis in Europe [21].

The difference between the cgMLST and wgMLST is that unlike cgMLST, wgMLST indexes the variation of pre-defined set of genes from both core and accessory genes [22]. Another retrospective study on 8 different outbreaks associated with verotoxigenic *Escherichia coli* (VTEC) O157:H7 in Canada showed that wgMLST provided higher discrimination than PFGE and MLVA [23].

Research studies have shown that cgMLST and wgMLST are viable typing methods for outbreak surveillance. In our study, cgMLST proved to provide higher discriminatory resolution for differentiating *Salmonella* Dublin isolates of outbreak group from the non-outbreak group. However, both cgMLST and wgMLST were unsuccessful in differentiating outbreak-related *Salmonella* Typhimurium isolates from outbreak-unrelated isolates.

Bacterial genome comprises a considerable amount (10 to 20%) of prophages integrated in their core genome [24]. Prophages harbor genes for antimicrobial resistance, virulence and toxins which contribute to the genetic diversity of bacterial strains making prophages a potential marker for discriminating *Salmonella* serovars [25]. However, one of the limitations of using prophage sequence profiles for *Salmonella* subtyping is the sensitivity and accuracy of the assembly as some prophage regions might be lost during assembly. We used two different *denovo* assemblers (SPAdes and Velvet) and found that prophage sequence profiling could not differentiate between the outbreak and non-outbreak isolates.

Recent studies have suggested that high throughput CRISPR typing has the potential to be used for epidemiological surveillance and investigation of *Salmonella* outbreaks [26, 27]. However, in our study, we detected identical spacers among outbreak and non-outbreak associated strains indicating that CRISPR typing is not useful for the surveillance of *Salmonella enetrica* outbreaks as we showed in our previous studies [28, 29] however, it might be useful for the discrimination among different *Salmonella* serovars.

Plasmid profiles and antimicrobial- susceptibility profiling have been used as an epidemiological tool since many decades. However, it was reported that analysis of plasmid profiles provided higher discrimination in the outbreak investigations than analysis of antimicrobial-susceptibility pattern [30, 31]. In our study both plasmid typing and in silico analysis of antibiotic resistance were unable to discriminate between the outbreak isolates and non-outbreak isolates.

In this study, we compared several retrospective WGS-based subtyping methods and we showed that SNP-based cluster analysis is superior to other subtying methods to define the source of outbreak in real-time.

In conclusion, foodborne salmonellosis is an important concern for public health therefore, it is crucial to use accurate, reliable and highly discriminative subtyping methods for epidemiological surveillance and outbreak investigation. The rapid development of next-generation sequencing (NGS) technology and bioinformatics tools have enabled WGS of any bacterial strain feasible. Various typing tools have been proposed by using WGS data but currently, the adoption of WGS-based methods have proved to be difficult due to lack of standardization. There are many layers on obtaining WGS data and there is need of standardization from the type of sequencers used to the bioinformatics analysis. Therefore, the emerging genetic analysis techniques should be combined with conventional phenotypic and molecular methods for routine surveillance and outbreak investigation until the WGS-based methods can be fully exploited, improved and standardized.

## Supplementary information

**Additional file 1: Supplementary Table 1.** Details of *Salmonella* Dublin strains analysed in this study. **Supplementary Table 2.** Details of *Salmonella* Typhimurium strains analysed in this study

## Data Availability

Available in supplementary Tables 1 and 2.

## Abbreviations

CGE: Centre for Genomic Epidemiology
cgMLST: Core genome multilocus sequence typing
CRISPRs: Clustered regularly interspaced short palindromic repeats
iNTS: Invasive NTS
ML: Maximum likelihood
MLST: Multilocus sequence typing
MLVA: Multiple loci VNTR analysis
NGS: Next generation sequencing
NTS: Non-typhoidal *Salmonella*
PE: Paired end
PFGE: Pulsed field gel electrophoresis
rMLST: Ribosomal MLST
SNP: Single nucleotide polymorphism
wgMLST: Whole genome MLST
WGS: Whole genome sequence
