# Supplementary material for: Evaluation of WGS-subtyping methods for epidemiological surveillance of foodborne salmonellosis
Source: One Health Outlook. 2020 Jul 6;2:13. doi: 10.1186/s42522-020-00016-5 (PMC7993512; doi:10.1186/s42522-020-00016-5)
Supplement: Supplementary file 1 — Additional file 1: Supplementary Table 1. Details of Salmonella Dublin strains analysed in this study. Supplementary Table 2. Details of Salmonella Typhimurium strains analysed in this study [file 42522_2020_16_MOESM1_ESM.docx]

**Supplementary table (1): Details of *Salmonella* Dublin strains analysed in this study:**

| **Strain ID** | **Isolation Year** | **Source (Human)** | **Accession number (Enterobase)** |
| --- | --- | --- | --- |
| **Outbreak strains:** | | | |
| **902637** | 2013 | Faeces | traces-0aDGEHd |
| **MF036933** | 2013 | Faeces | traces-0tThsgT |
| **MF036980** | 2013 | Faeces | traces-0Brillc |
| **517138** | 2013 | Faeces | traces-0uulMcg |
| **MF6869** | 2013 | Faeces | traces-0MrnpnH |
| **M26560** | 2013 | Faeces | traces-0RqTIyQ |
| **MF7067** | 2013 | Faeces | traces-0FppneO |
| **MF7174** | 2013 | Faeces | traces-0tNyHNf |
| **40986** | 2013 | Faeces | traces-0jbNvsG |
| **Non-outbreak strains:** | | | |
| **MF038630** | 2013 | Faeces | traces-0YdbXxL |
| **M1314220** | 2013 | Blood | traces-0pdYwFJ |
| **M54827** | 2012 | Blood | traces-0gpCZvN |
| **MB12371** | 2012 | Blood | traces-0OKAAKN |
| **MF5994** | 2012 | Faeces | traces-0pFGzCD |
| **MB7978** | 2012 | Blood | traces-0KFJ0jx |
| **B289223** | 2012 | Blood | traces-0Ufzknp |
| **11F310** | 2011 | Faeces | traces-0Fheffj |
| **MB98550** | 2010 | Blood | traces-0DwCeOd |
| **MF8409** | 2010 | Faeces | traces-0uGHvve |
| **W151R0** | 2010 | Psoas abscess | traces-0AzQdTx |
| **B261193** | 2010 | Blood | traces-0NRFiqK |
| **MP015199F** | 2010 | Urine | traces-0OvBUlw |
| **Food strains:** | | | |
| 2014LSAL02972 | 2014 | St Nectaire cheese | traces-0wDMlBa |
| 2015LSAL00258 | 2015 | Morbier cheese | traces-0UpFAuq |

**Supplementary table (2): Details of *Salmonella* Typhimurium strains analysed in this study**

| **Strain ID** | **Receipt date** | **Source** | **ENA Accession number** | **Accession number (Enterobase):** |
| --- | --- | --- | --- | --- |
| **Food strains:** | | | | |
| **H133060375** | 25/07/2013 | Mayonnaise | SRR2163444 | traces-0jBkjNE |
| **H133060376** | 25/07/2013 | Mayonnaise | SRR2163445 | traces-0dGjRLW |
| **H133060377** | 25/07/2013 | Mayonnaise | SRR2163446 | traces-0OUwaTM |
| **H133060378** | 25/07/2013 | Mayonnaise | SRR2163488 | traces-0DBiaaH |
| **Outbreak strains:** | | | | |
| **H133000654** | 22/07/2013 | Human | SRR2163487 | traces-0yFVtEJ |
| **H132940743** | 17/07/2013 | Human | SRR2163469 | traces-0KhTEXU |
| **H132940744** | 17/07/2013 | Human | SRR2163437 | traces-0IPDGvh |
| **H132940745** | 17/07/2013 | Human | SRR2163440 | traces-0VRsUCN |
| **H132940746** | 17/07/2013 | Human | SRR2163471 | traces-0FueXxu |
| **H132940748** | 17/07/2013 | Human | SRR2163458 | traces-0VQoRAI |
| **H132940749** | 17/07/2013 | Human | SRR2163465 | traces-0lXtqHW |
| **H132940750** | 17/07/2013 | Human | SRR2163442 | traces-0dtaLTL |
| **H132940751** | 17/07/2013 | Human | SRR2163436 | traces-0peCZhu |
| **H132940753** | 17/07/2013 | Human | SRR2163434 | traces-0jsnddC |
| **H132940754** | 17/07/2013 | Human | SRR2163459 | traces-0oesFol |
| **H132940756** | 17/07/2013 | Human | SRR2163484 | traces-0cXyjGF |
| **H133000645** | 22/07/2013 | Human | SRR2163480 | traces-0MptDPD |
| **H133300609** | 12/08/203 | Human | SRR2163438 | traces-0TWepNb |
| **H132300541** | 03/06/2013 | Human | SRR2163435 | traces-0HQZVLf |
| **Non-outbreak strains:** | | | | |
| **H133260293** | 08/08/2013 | Human | SRR2163486 | traces-0CoTGFn |
| **H132780266** | 05/07/2013 | Human | SRR2163433 | traces-0uLjdHA |
| **H132960590** | 18/07/2013 | Human | SRR2163456 | traces-0QbJMrF |
| **H132920685** | 16/07/2013 | Human | SRR2163474 | traces-0YYssib |
| **H132980531** | 19/07/2013 | Human | SRR2163489 | traces-0SzSijx |
| **H121600325** | 16/04/2012 | Human | SRR1635127 | traces-0hhCVRQ |
| **H122720573** | 03/07/2012 | Human | SRR1645461 | traces-0VxyMld |
| **H12320661** | 25/09/2012 | Human | SRR1645554 | traces-0UJrUip |
| **H123020544** | 24/07/2012 | Human | SRR1645246 | traces-0VJgsMn |
| **H132020501** | 14/05/2013 | Human | Srr2163450 | traces-0sMnwms |
| **H122020454** | 15/05/2012 | Human | SRR1646143 | traces-0oVfhNe |
| **H124860455** | 29/11/2012 | Human | SRR1645387 | traces-0gFaInb |
| **H133040470** | 24/07/2013 | Human | SRR2163462 | traces-0JTAKFm |
| **H1330400611** | 19/08/2013 | Human | SRR2163457 | traces-0ghtsQf |
